# A New Feature Ensemble with a Multistage Classification Scheme for Breast Cancer Diagnosis

**DOI:** 10.1155/2017/3895164

**Published:** 2017-06-19

**Authors:** Idil Isikli Esener, Semih Ergin, Tolga Yuksel

**Affiliations:** ^1^Department of Electrical Electronics Engineering, Bilecik Seyh Edebali University, 11210 Bilecik, Turkey; ^2^Department of Electrical Electronics Engineering, Eskisehir Osmangazi University, 26480 Eskisehir, Turkey

## Abstract

A new and effective feature ensemble with a multistage classification is proposed to be implemented in a computer-aided diagnosis (CAD) system for breast cancer diagnosis. A publicly available mammogram image dataset collected during the Image Retrieval in Medical Applications (IRMA) project is utilized to verify the suggested feature ensemble and multistage classification. In achieving the CAD system, feature extraction is performed on the mammogram region of interest (ROI) images which are preprocessed by applying a histogram equalization followed by a nonlocal means filtering. The proposed feature ensemble is formed by concatenating the local configuration pattern-based, statistical, and frequency domain features. The classification process of these features is implemented in three cases: a one-stage study, a two-stage study, and a three-stage study. Eight well-known classifiers are used in all cases of this multistage classification scheme. Additionally, the results of the classifiers that provide the top three performances are combined via a majority voting technique to improve the recognition accuracy on both two- and three-stage studies. A maximum of 85.47%, 88.79%, and 93.52% classification accuracies are attained by the one-, two-, and three-stage studies, respectively. The proposed multistage classification scheme is more effective than the single-stage classification for breast cancer diagnosis.

## 1. Introduction

Cancer is a group of body cells that grow and proliferate abnormally and uncontrollably because of damaged DNA (deoxyribonucleic acid). This group of body cells, known as tumors, may be either benign or malignant. Benign tumors are not cancerous and life-threatening as they do not spread to other tissues or organs of the body. In stark contrast to benign tumors, malignant ones tend to be metastasized and may generally be fatal.

Breast cancer originates in a breast tissue. It is the most frequently diagnosed cancer among women, and it is 100 times more common in women than in men [1]. Worldwide, breast cancer is the second major cause of female deaths resulting from cancer [2]. There is no known way to prevent breast cancer, but mortality can be reduced with early diagnosis [3]. Radiological screening is the most important action to take for early diagnosis [4]. Although mammography is known as the most effective radiological screening technique both for breast investigation and diagnosis, the subtle difference of X-ray permeability between normal and abnormal regions makes cancer detection difficult [5]. This difficulty is aggravated as the breast tissue type becomes denser. Moreover, human factors heavily affect the interpretation of mammogram images. A computer-aided diagnosis (CAD) system detects and diagnoses cancer without these negative factors [6]. Hence, using a CAD system increases the sensitivity of cancer detection by providing radiologists a second opinion.

Classification accuracy of CAD systems is directly affected by detection of suspicious regions for breast cancer, namely region of interest (ROI), from whole-breast mammogram images. Besides the low-contrast problem, the digitization noise in mammograms also affects the success of ROI detection negatively; and noise reduction is required to improve the image quality [7, 8]. Hence, preprocessing is necessary and should be the first of the four stages in a CAD system. Some studies have tried to overcome the problem of low contrast using histogram processing operations [8–11], morphological operations [12], and statistics theory [13], while unsharp filtering [8], wavelet transform [12, 13], and median filtering [14, 15] are the most common noise reduction.

In the second stage of a CAD system, the ROIs are detected from entire breast images. ROI detection in the past decade was generally performed using wavelet transforms [16], segmentation algorithms [17, 18], and edge operators [19].

The efficiency of a CAD system is directly related to the efficacy of data representation. Feature extraction, which is the third stage, is an undoubtedly important task for pattern recognition and is implemented with a remarkable number of techniques in several studies. Specifically, there are statistical techniques [20–25], model-based techniques [26, 27], graph-theoretic approaches [28], and signal processing techniques that compute breast tissue features from pixel characteristics [22, 29] or frequency spectrum [21, 23, 24, 30–32] for breast cancer diagnosis on a mammographic image. Additionally, there are various studies using mammographic features [20, 33–36] like shape, spicule index, contour, size, density, and brightness.

Finally, in the fourth stage, extracted discriminative features are used for the classification of ROIs into normal, benign, and malignant lesions. Artificial neural networks [20, 27, 29, 36, 37], support vector machines (SVMs) [20, 25, 30, 31, 33, 38, 39], subspace learning algorithms [22, 25, 38, 39], Bayes, decision tree, and k-nearest neighbor classifiers [20, 25] are well-known classifiers used for mammogram classification.

Mammogram-based breast cancer diagnosis studies can be categorized as microcalcification detection, mass detection, and mass recognition. Pal et al. presented a multistage system for microcalcification detection [27]. This multistage system first classifies a mammogram image as normal or abnormal; then, for an abnormal image, it detects the regions with microcalcification. The authors extracted statistical features on manually detected ROIs and implemented feature selection and classification using a multilayer perceptron neural network [27]. Lado et al. developed an extended generalized additive model (GAM) involving interaction of breast tissue factors to reduce the false-positive rate for microcalcification detection [16]. The authors stated that the false-positive rate has decreased to 0.74 per image from 1.46 when the breast tissue type is integrated into the GAM. Similarly, Malar et al. studied the effectiveness of breast tissue type integration on microcalcification detection using an extreme learning machine and achieved an accuracy of 94% using wavelet-based features [40]. Since the number of cells with microcalcification is smaller than the number of healthy cells, microcalcification detection is an unbalanced classification problem. Bria et al. proposed a cascaded five-classifier approach to eliminate the predominance of healthy cells [41]. In this approach, the first classifier initially discriminates the normal and abnormal cells and later benign microcalcification clusters (*μ*Cs) and false detections of normal cells are eliminated using a RankBoost classifier. The resultant malignant *μ*Cs are evaluated by the next classifier, and the process goes on until the *μ*Cs from the last classifier are obtained. Ultimately, final *μ*Cs are selected according to their probability maps with 93% accuracy. Kekre et al. [17, 18] segmented mammogram images using a vector quantization technique for mass detection. They computed the areas of each region on the segmented images and classified the region having the largest area as a mass. Hachama et al. used an image registration for mass detection [42]. Savitha et al. suggested that analyzing mammogram images in the complex plane will increase the accuracy of mass detection [43]. They mapped the mammogram images into a complex plane and classified them using a fully complex-valued relaxation neural network with an accuracy of 97.84%. Vallez et al. stated that lesion detection and recognition accuracy can be increased by using predefined breast tissue type information [25]. The classification accuracy rate has been increased to 91% from 78% in their study. Guliato et al. suggested that the previously proposed polygonal modeling [44] is an effective method for mammogram classification as it helps in noise reduction while preserving the important features [45]. Oliver et al. proposed a knowledge-based approach for the automatic detection of microcalcifications and clusters in mammographic images [37]. In this approach, local features that characterize the morphology of microcalcifications are first extracted to create a dictionary of visual words by a bank of filters. Then, feature selection is accomplished by using a boosted classifier for microcalcification detection. Finally, the cluster detection is achieved at 80% sensitivity by locally integrating the individual microcalcification probability images.

In this paper, a new and effective feature ensemble with a multistage classification is proposed to be implemented in a CAD system for breast cancer diagnosis. The result is verified using a publicly available mammogram image dataset collected during the Image Retrieval in Medical Applications (IRMA) project. For the preprocessing stages, contrast enhancement and noise reduction operations are first executed on each mammogram ROI in the database by applying a histogram equalization followed by a nonlocal means (NLM) filtering [46]. The local configuration pattern (LCP) algorithm [47] is then applied to obtain LCP-based feature vectors from the mammographic images. Then, some statistical and frequency-domain features are extracted and concatenated with the LCP-based feature vectors. Eventually, these feature vectors formed by LCP-based, statistical, and frequency-domain features are classified as normal, benign, and malignant using eight different popular classifiers via cross validation. The classification process is performed in three different cases in this study. In the first case, called a one-stage study, the feature vectors are directly classified into three classes. In the second case, called a two-stage study, the feature vectors are initially categorized according to their breast tissue types, and are subsequently classified as normal, benign, and malignant. In the third case, called a three-stage study, the feature vectors are first classified according to their breast tissue types. Afterward, they are classified as normal and abnormal. At the third stage of this case, the feature vectors labeled as abnormal classes are categorized as benign and malignant. Moreover, a classifier combination via a majority voting of the most successful three classifiers is employed for both the two- and three-stage studies.

This paper is organized as follows. The preprocessing and the whole feature extraction procedure realized in this paper are explicated and all of the classification methods and the evaluation metrics are briefly described in the following section. Discussions on the experimental studies and the obtained results are given in Section 3, whereas the main conclusions are precisely specified in the last section.

## 2. Materials and Methods

### 2.1. Database

It is very important to work on images with their ground truths for medical imaging applications [48]. In this study, a publicly available mammogram dataset constructed during the IRMA project is used [49]. This dataset consists of 12 classes defined by the Breast Imaging Reporting and Data System (BI-RADS). There are four breast tissue classes (fatty, fibroglandular, heterogeneously dense, and extremely dense) and three health status classes (normal, benign tumor, and malignant tumor) for each breast tissue type. There are 233 mammogram ROI parts, lower-dimensional mammogram images that consist of just healthy/cancerous regions of the whole breast, for each class, and therefore, a total of 2796 parts are available in the dataset [49]. The ROI parts of each class are classified using cross-validation technique. It implicitly means that 210 of 233 parts (90%) in each class are used for training while the remaining 23 of 233 parts (10%) are treated as the test parts. The process is repeated for each fold in the cross-validation technique, and the average classification accuracy for each classifier is obtained.

### 2.2. Preprocessing

In the preprocessing stage, a histogram equalization followed by the NLM filtering is applied on the mammogram parts [48]. The NLM filter is an adaptive smoothing filter that changes the window size according to the similarity between neighborhoods of any two pixels as well as preserves the fine details by computing a weighting function according to the derivatives in the corresponding search window [46, 48]. Given a discrete noisy image *v* = {*v*(*i*)|*i* ∈ *I*}, the filtered value NL[*v*(*i*)] of any pixel is computed as

(1) $$\begin{array}{c} NL\left[v\left(i\right)\right]=\underset{j\in I}{\sum }w\left(i,j\right)\cdot v\left(j\right), \end{array}$$

where *w*(*i*, *j*) refers to the weight coefficient computed utilizing the similarity between pixels *i* and *j* and satisfies the conditions 0 ≤ *w*(*i*, *j*) ≤ 1 and ∑_*j*_*w*(*i*, *j*) = 1.

The similarity between pixels *i* and *j* is measured as the Gaussian weighted Euclidean distance, ‖*v*(*N*_*i*_) − *v*(*N*_*j*_)‖_2,*σ*_^2^, where *σ*  (*σ* > 0) is the standard deviation of the Gaussian kernel, whereas *v*(*N*_*i*_) and *v*(*N*_*j*_) are the neighborhoods of pixels *i* and *j* in the similarity window [48]. The pixels with larger weights indicate a similar neighborhood as it can be understood by analyzing (2). *Z*_*i*_ and *h* in (2) refer to the normalizing constant and the degree of filtering, respectively.

(2) $$\begin{array}{c} w\left(i,j\right)=\frac{1}{Z_{i}}\cdot e^{-{\left\|v\left(N_{i}\right)-v\left(N_{j}\right)\right\|}_{2,\sigma }^{2}/h^{2}}, \end{array}$$

(3) $$\begin{array}{c} Z_{i}=\underset{j}{\sum }e^{-{\left\|v\left(N_{i}\right)-v\left(N_{j}\right)\right\|}_{2,\sigma }^{2}/h^{2}}. \end{array}$$

### 2.3. Feature Extraction

The most essential stage in CAD systems, as well as in any pattern recognition problem, is the feature extraction in which data is represented in a low-dimensional space by the most descriptive features that maximize and characterize the interclass differences. In this study, three groups of features are concatenated to construct the feature vectors. The first group is LCP-based features obtained using LCP algorithm, while the second and third groups are some statistical and frequency-domain features, respectively.

#### 2.3.1. Local Configuration Pattern

The local binary pattern (LBP) is generally used for face representation and recognition in the past two decades [50–52], and it is a grayscale and rotation-invariant feature extraction technique presented by Ojala et al. [53].

The grayscale-independent LBP representation of an image *I* is obtained by thresholding *P* neighbors in the circular neighborhood of radius *R* with the intensity value of the central pixel as given in (4).

(4) $$\begin{array}{c} LBP\left(P,R\right)=\overset{P-1}{\underset{i=0}{\sum }}u\left(g_{i}-g_{c}\right)\cdot 2^{i}, \end{array}$$

(5) $$\begin{array}{c} u\left(x\right)=\left\{\begin{array}{ll} 1, & x\geq 0\\ 0, & x<0. \end{array}\right. \end{array}$$

The terms *g*_*i*_ and *g*_*c*_ in (4) denote the intensity values of the neighboring pixel *i* and central pixel *c*, respectively. The rotation-invariant LBP-based feature vectors are described by the idea of rotating each bit pattern circularly to a minimum value ending up with the maximum value as the last element of the feature vectors. Equation (6) introduces the mathematical representation of this idea where the term LBP^riu2^ refers to the rotation-invariant LBP-based feature vectors.

(6) $$\begin{array}{c} {LBP}^{riu2}\left(P,R\right)=\left\{\begin{array}{ll} \overset{P-1}{\underset{i=0}{\sum }}u\left(g_{i}-g_{c}\right), & U\left(LBP\left(P,R\right)\right)\leq 2\\ P+1, & otherwise. \end{array}\right. \end{array}$$

The quantization of gray-level differences to binary levels sometimes causes undesirably the same LBP representations although the neighborhoods are relatively different. This problem is solved by computing the local variance (VAR) of each pattern, and the joint histogram (*O*) is formed. *μ* in (7) refers to the average intensity of the neighboring pixels.

(7) $$\begin{array}{c} VAR=\frac{1}{P}\;\overset{P-1}{\underset{i=0}{\sum }}{\left(g_{i}-\mu \right)}^{2}, \end{array}$$

(8) $$\begin{array}{c} O=\frac{{LBP}^{riu2}}{VAR}. \end{array}$$

The LBP algorithm is stated to be an effective technique for detecting local structures; however, the LBP^riu2^ feature vectors for patterns having equal variances may be the same although they have different configurations [47]. Guo et al. proposed a microscopic (MiC) descriptor that defines the microscopic configuration of an image by a linear configuration model as a solution to this problem [47]. In this model, the optimal weights (*A*_L_) of the neighboring pixels are calculated via the least square estimation technique to form the central pixel. For the conservation of being a rotationally invariant characteristic, a one-dimensional Fourier transform of optimal weight vectors is computed and *H*_L_ values are obtained. The magnitude of *H*_L_ is defined as the MiC feature of a pattern.

The local configuration pattern (LCP) is a technique that describes the local structures and microscopic configuration of a pattern together, where the LCP-based feature vector of an image is obtained by concatenating the microscopic configuration of each pattern in an image with their joint histogram as [24]

(9) $$\begin{array}{c} LCP=\left[\left[\left.H_{0}\right|;O_{0}\right];\left[\left.H_{1}\right|;O_{1}\right];\ldots ;\left[\left.H_{q-1}\right|;O_{q-1}\right]\right], \end{array}$$

where *q* is the number of patterns in an image.

#### 2.3.2. Statistical Features

Some significant and descriptive statistical features of each LCP-based feature vector are calculated as the second group of features to increase the data representability of the feature vectors. Energy is one of the most important statistical features of any distribution, and hence, the energy values of LCP-based feature vectors are evaluated. The mean, maximum, minimum, and mean energy of each LCP-based feature vector are additionally computed as statistical features. In the statistical theory, the variance, skewness, and kurtosis are defined as variation criterions. Owing to the large variations between healthy and cancerous regions on a mammogram image, these criterions are also calculated. Moreover, the standard deviation, energy variance, and area descriptor [54] of LCP-based feature vectors are additional variation-related features used in this study. Radiologists state that cancerous regions and malignant regions have more irregular distribution than healthy regions and benign regions, respectively. This statement corresponds to entropy in statistics. Therefore, the entropy of each LCP-based feature vector is calculated to measure this irregularity as a feature. The statistical features utilized in this study and their mathematical representations for the *N* × 1 dimensional feature vectors are listed in Table 1.

#### 2.3.3. Frequency-Domain Features

The third group of features computed in this study is the frequency-domain features. Frequency-domain features are determined by applying a two-level two-dimensional discrete wavelet transform (2D-DWT) using Daubechies1 (db1) wavelet function on the preprocessed mammogram images, and finally, 16 sub-bands for each mammogram image are obtained. The energy values of each sub-band are computed since the brightness is one of the most significant issues for breast cancer diagnosis. db1 function is a type wavelet in wavelet analysis. The mother function *ψ*(*t*) of db1 wavelet is described as [55]

(10) $$\begin{array}{c} \psi \left(t\right)=\left\{\begin{array}{ll} 1, & 0\leq t<\frac{1}{2}\\ 1, & \frac{1}{2}\leq t<1\\ 0, & otherwise. \end{array}\right. \end{array}$$

#### 2.3.4. Feature Vector Construction

The preprocessed mammogram parts are decomposed into four sub-bands that are LL (low-low), LH (low-high), HL (high-low), and HH (high-high) by a one-level 2D-DWT utilizing the db1 wavelet. Several parameter values are experienced in the LCP transform, and ultimately, the LCP algorithm is applied on each sub-band using 8 neighbors in the circular neighborhood of radius 2. Therefore, 81 × 1 dimensional LCP vectors of each sub-band are constructed. The endmost values in those LCP vectors are appreciably high; therefore, they are removed to get rid of their domination over other features. The remaining 80-dimensional feature vectors of each sub-band {LL‐LH‐HL‐HH} are then weighted with the respective coefficients {1.4‐1‐1‐0} concluded as the most efficient coefficients by [5]. Then, they are summed up to form an 80-dimensional feature vector for each mammogram part [48].

In order to increase the representative power of the feature vectors, 12 statistical features computed from the LCP-based feature vectors, and 16 frequency-domain features evaluated from the sub-bands obtained by the decomposition of the preprocessed mammogram ROI parts using the two-level 2D-DWT are concatenated to the LCP-based feature vectors [48]. Consequently, 108-dimensional feature vectors are extracted from each ROI part. The statistical features are extracted from the LCP-based feature vectors instead of extracting them directly from the mammogram texture to amplify the discriminative power of the LCP-based feature vectors. The frequency-domain features, which are the energy values of each sub-band in the spatial domain, are extracted since the brightness is one of the most significant issues for breast cancer diagnosis, and changes in the brightness in a mammogram image are clearly observed in the spatial frequency.  Table 2 summarizes the feature vector construction process. In Table 2, the phrase “LCP: energy” refers to the energy value of an LCP vector whereas “LLLL: energy” is the energy of the LLLL (low-low-low-low) sub-band.

### 2.4. Classifiers

#### 2.4.1. Fisher's Linear Discriminant Analysis

Fisher's linear discriminant analysis (FLDA) tries to find a projection matrix that projects the training data onto a low-dimensional space that maximizes between-class variance as well as minimizing within-class variance [48, 56]. This is known as the Fisher maximization criterion and is defined as

(11) $$\begin{array}{c} J\left(\vec{w}\right)=\frac{{\vec{w}}^{T}\cdot S_{B}\cdot \vec{w}}{{\vec{w}}^{T}\cdot S_{W}\cdot \vec{w}}, \end{array}$$

where $\vec{w}$, *S*_B_, and *S*_W_ refer to the projection vectors and between-class and within-class scatter matrices, respectively.

On the test stage of FLDA, any test vector is projected via $\vec{w}$ projection vectors, and distances to the training vectors on the low-dimensional space are calculated [48]. The decision criterion for FLDA is given as

(12) $$\begin{array}{c} K=\underset{1\leq c\leq S}{\arg\;\min}\left\{\;\left\|{\vec{\Omega }}^{c}-{\vec{\Omega }}_{test}\right\|\;\right\}, \end{array}$$

where *c* is the class index, *S* is the total number of classes, and ${\vec{\Omega }}^{c}$ and ${\vec{\Omega }}_{test}$ are the projected training vector of the *c*th class and the projected test vector, respectively [48].

#### 2.4.2. Linear Discriminant Classifier

Linear discriminant classifier (LDC) tries to find the weight vectors $\vec{w}$ of a linear hyperplane $g\left(\vec{x}\right)$ that separates given classes [57]. The weight vectors of this hyperplane are defined by a linear combination of training feature vectors $\left(\vec{x}\right)$ of each class. The linear hyperplane is characterized by the weight vectors and a threshold *w*_0_ as

(13) $$\begin{array}{c} g\left(\vec{x}\right)={\vec{w}}^{T}\cdot \vec{x}+w_{0}. \end{array}$$

The LDC assigns any test vector $\left({\vec{x}}_{test}\right)$ to a class according to the sign of the projection function given in (14) for a two-class problem. The terms *w*_1_ and *w*_2_ in (14) refer to the class labels.

(14) $$\begin{array}{c} {\vec{x}}_{test}\in \left\{\begin{array}{ll} w_{1}, & {\vec{w}}^{T}\cdot {\vec{x}}_{test}+w_{0}>0\\ w_{2}, & {\vec{w}}^{T}\cdot {\vec{x}}_{test}+w_{0}<0. \end{array}\right. \end{array}$$

#### 2.4.3. Support Vector Machines

Support vector machines (SVMs), also known as maximum margin classifiers, determine the optimal hyperplane that maximizes the distance between the hyperplane and support vectors [58]. Support vectors are the training vectors that are nearest from each class to the hyperplane [59]. As it can classify linearly separable data, SVM can classify nonlinear data by transforming the data to a higher-dimensional space by using an appropriate kernel function [49]. If the training set is $TS=\left\{\left({\vec{x}}_{1},L_{1}\right),\left({\vec{x}}_{2},L_{2}\right),\ldots ,\left({\vec{x}}_{M},L_{M}\right)\right\}$ for a two-class problem, where ${\vec{x}}_{i}\;\left(i=1,2,\ldots ,M\right)$ is the training data and *L*_*i*_  (*L*_*i*_ ∈ {−1, 1}) is the class label, the test vector is classified according to the sign of the function given as

(15) $$\begin{array}{c} f\left({\vec{x}}_{test}\right)=\sum \left\{\;\alpha_{i}\cdot L_{i}\cdot \left({\vec{x}}_{i}^{T}\cdot {\vec{x}}_{test}\right)+b\;\right\}, \end{array}$$

where *α*_*i*_  (*i* = 1, 2,…, *M*) are the nonzero quadratic coefficients and $\left(\left|b\right|/\left\|\vec{w}\right\|\right)$ is the perpendicular distance between the hyperplane and the origin, whereas $\vec{w}$ is the normal vector of the separating hyperplane [48].

#### 2.4.4. Logistic Linear Classifier

The logistic linear classifier (LLC) states that a linear hyperplane can be characterized by the relationship between the dependent and independent variables of training feature vectors $\left(\vec{x}\right)$ [60]. In LLC, this relationship is determined using a logistic regression analysis by computing class-conditional probability density functions of $\vec{x}$ vectors. The LLC model for a two-class problem is given by (16) where $p\left(\left.\vec{x}\right|w_{i}\right)$, $\vec{\beta }$, and *β*_0_ are the class-conditional probability density functions of $\vec{x}$, weight vectors for the linear hyperplane, and a threshold value, respectively.

(16) $$\begin{array}{c} \log\;\left(\frac{p\left(\left.\vec{x}\right|w_{1}\right)}{p\left(\left.\vec{x}\right|w_{2}\right)}\right)={\vec{\beta }}^{T}\cdot \vec{x}+\beta_{0}. \end{array}$$

The LLC assumes that log-linear models can be formed between classes with equal prior probabilities and covariance matrices. This assumption is equivalent to

(17) $$\begin{array}{c} \begin{array}{l} p\left(\left.w_{1}\right|\vec{x}\right)=\frac{\exp\left({\vec{\beta }}^{T}\cdot \vec{x}+\beta_{0}^{'}\right)}{1+\exp\left({\vec{\beta }}^{T}\cdot \vec{x}+\beta_{0}^{'}\right)},\\ p\left(\left.w_{2}\right|\vec{x}\right)=\frac{1}{1+\exp\left({\vec{\beta }}^{T}\cdot \vec{x}+\beta_{0}^{'}\right)}, \end{array} \end{array}$$

(18) $$\begin{array}{c} \beta_{0}^{'}=\beta_{0}+\log\;\left(\frac{p\left(w_{1}\right)}{p\left(w_{2}\right)}\right), \end{array}$$

where $p\left(\left.w_{i}\right|\vec{x}\right)$ and *p*(*w*_*i*_) are the probabilities of class *w*_*i*_ given $\vec{x}$ and prior probability of class *w*_*i*_, respectively. The decision criterion for LLC is given in

(19) $$\begin{array}{c} \vec{x}\in \left\{\begin{array}{ll} w_{1}, & \frac{p\left(\left.w_{1}\right|\vec{x}\right)}{p\left(\left.w_{2}\right|\vec{x}\right)}>1\\ w_{2}, & \frac{p\left(\left.w_{1}\right|\vec{x}\right)}{p\left(\left.w_{2}\right|\vec{x}\right)}<1, \end{array}\right. \end{array}$$

(20) $$\begin{array}{c} \vec{x}\in \left\{\begin{array}{ll} w_{1}, & {\vec{\beta }}^{T}\cdot \vec{x}+\beta_{0}^{'}>0\\ w_{2}, & {\vec{\beta }}^{T}\cdot \vec{x}+\beta_{0}^{'}<0. \end{array}\right. \end{array}$$

#### 2.4.5. Decision Tree

The principle of the decision tree classifier is to cluster any data into subgroups until all elements of any subgroup have the same class label [48, 61]. Classification rules are defined by clustering the data into the leaves, class labels, in the training stage while those rules are applied to any test sample and the leaf that the test sample reaches provides the class label of the test sample in the test stage.

#### 2.4.6. Random Forest

The random forest classifier is an ensemble of decision tree classifiers developed to improve the classification accuracy [62]. Each tree classifier in this ensemble votes for the best class of any sample, and the resultant class label is then specified via a majority voting technique.

#### 2.4.7. Naïve Bayes

Bayesian classifiers compute the probability of each class given any test vector $\left(\vec{x}\right)$ and assign it to the class with the highest conditional probability [63]. The Bayesian decision criterion for a two-class problem is

(21) $$\begin{array}{c} P\left[\left.w_{1}\right|\vec{x}\right]>P\left[\left.w_{2}\right|\vec{x}\right]\Leftrightarrow \vec{x}\in w_{1}. \end{array}$$

The terms $P\left[\left.w_{1}\right|\vec{x}\right]$ and $P\left[\left.w_{2}\right|\vec{x}\right]$ denote the posterior probabilities of classes *w*_1_ and *w*_2_ given $\overset{\to }{x}$, respectively, where $P\left(\left.w_{i}\right|\vec{x}\right)$ is computed as

(22) $$\begin{array}{c} P\left(\left.w_{i}\right|\vec{x}\right)=\frac{p\left(\left.\vec{x}\right|w_{i}\right)\cdot P\left(w_{i}\right)}{p\left(\vec{x}\right)}, \end{array}$$

(23) $$\begin{array}{c} p\left(\vec{x}\right)=\overset{2}{\underset{i=1}{\sum }}p\left(\left.\vec{x}\right|w_{i}\right)\cdot P\left(w_{i}\right). \end{array}$$

The terms *P*(*w*_*i*_), $p\left(\left.\vec{x}\right|w_{i}\right)$, and $p\left(\vec{x}\right)$ refer to the prior probability of class *w*_*i*_, the probability of $\vec{x}$ given class *w*_*i*_, and the probability density function of $\vec{x}$, respectively. One-dimensional and *l*-dimensional case computations of $p\left(\left.\vec{x}\right|w_{i}\right)$ are given in (24) and (25), respectively. *μ*, *σ*, and ∑ in these equations are the mean, variance, and covariance matrix of the feature vectors, respectively.

(24) $$\begin{array}{c} p\left(\left.\vec{x}\right|w_{i}\right)=\frac{1}{\sqrt{2\pi }\cdot \sigma }\cdot \exp\left(-\frac{{\left(\vec{x}-\mu \right)}^{2}}{2\sigma^{2}}\right), \end{array}$$

(25) $$\begin{array}{c} p\left(\left.\vec{x}\right|w_{i}\right)=\frac{1}{{\left(2\pi \right)}^{1/2}{\left|\sum \right|}^{1/2}}\cdot \exp\left(-\frac{1}{2}\cdot {\left(\vec{x}-\mu \right)}^{T}\cdot \sum^{-1}\cdot \left(\vec{x}-\mu \right)\right). \end{array}$$

Naïve Bayes classifiers assume that all feature vectors are statistically independent and classify any test vector according to the Bayesian decision criterion given in (21) [63]. In this classification scheme, the probability density function for the *l*-dimensional case is computed as

(26) $$\begin{array}{c} p\left(\vec{x}\right)=\overset{l}{\underset{i=1}{\prod }}p\left(x\left(i\right)\right). \end{array}$$

#### 2.4.8. *k*-Nearest Neighbors

The *k*-nearest neighbor (kNN) classifier assigns any test vector to the respective class that its *k*-nearest neighbors belong at most, considering the distances between the test and training vectors in the feature space [64]. Although it is obvious that classification performance is directly related to the parameter *k*, there is no obvious information on the selection of *k* except that it should be positive and not a multiple of the total number of classes [48].

### 2.5. Evaluation Metrics

The metrics sensitivity (SNS), specificity (SPC), positive predictive value (PPV), negative predictive value (NPV), false-positive rate (FPR), false-negative rate (FNR), false discovery rate (FDR), false omission rate (FOR), and accuracy (ACC) are used for the evaluation of the performance of the CAD system in this study. The mathematical representations of these metrics are given in Table 3.

## 3. Results and Discussion

**Figure 1:**
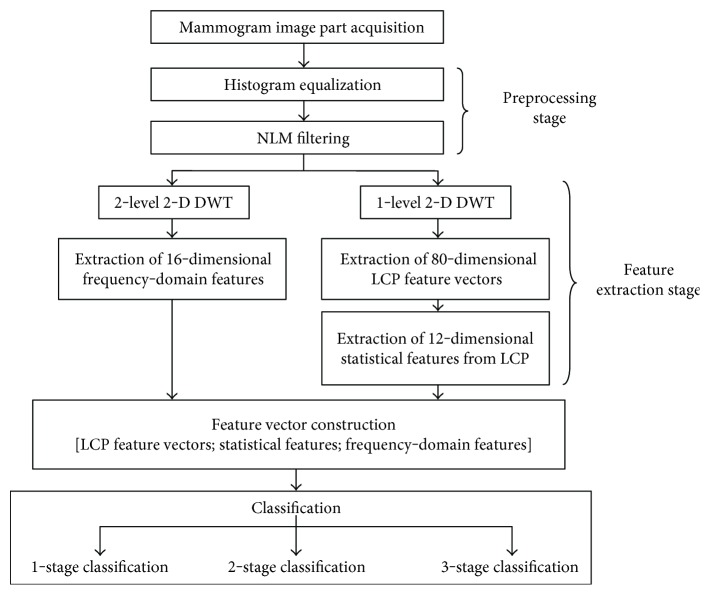
Block diagram of the proposed system.

In this study, a CAD system for breast cancer diagnosis based on a multistage classification using a novel feature ensemble is proposed. The feature extraction stage is achieved on mammogram ROIs that are preprocessed by applying a histogram equalization followed by the NLM filtering. The proposed feature ensemble is formed by concatenating the LCP-based, statistical, and frequency-domain features. The classification process of these features is implemented in three different cases: one-stage study, two-stage study, and three-stage study. The mammogram ROIs are classified into three classes (normal, benign, and malignant) regardless of their breast tissue types in the one-stage study while the two- and three-stage studies consider breast tissue information and make a health status classification as explicitly explained in the related subsections. Eight well-known classifiers (FLDA, LDC, linear SVM, LLC, decision tree, random forest, naïve Bayes, and kNN) are used in all of the classification cases. Additionally, the results of classifiers that show the top three performances are combined via a majority voting technique in order to improve the recognition accuracy for the both two- and three-stage studies. The block diagram of the proposed system is given in Figure 1.

### 3.1. Results

#### 3.1.1. One-Stage Study

**Figure 2:**
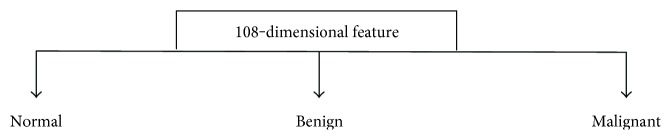
Flowchart designed for the one-stage study.

**Figure 3:**
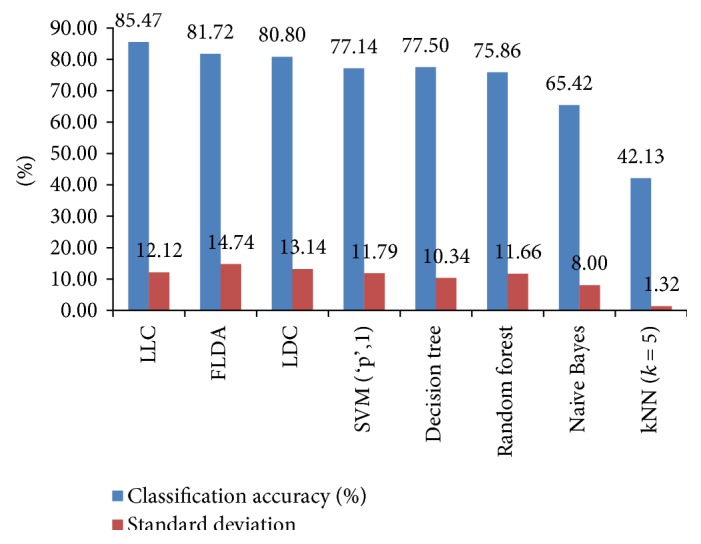
Average classification accuracies and standard deviations of eight different classifiers obtained by elevenfold cross-validation for the one-stage study.

In this case of the classification scheme, the feature vectors are directly classified into three classes (normal, benign, and malignant) regardless of the breast tissue types of the mammogram images. The flowchart for the one-stage study is shown in Figure 2. The average classification accuracies and standard deviations of the classifiers for the one-stage study obtained by elevenfold cross-validation technique are shown in Figure 3. In this figure, “SVM (‘p', 1)” is the SVM classifier using a linear kernel. The LLC classifier has the highest recognition accuracy (85.47%) among all classifiers. It assumes that logistic linear models can be formed between classes with equal prior probabilities. Hence, it is more applicable for the one-stage study than the other classifiers as the prior probabilities of each class in this case are equal.

The total confusion matrix of the LLC classifier obtained by elevenfold cross-validation for the one-stage study is given in Table 4. It shows that benign and malignant mammograms are distinguishable from each other. The false recognitions are caused by the confusion of the benign and malignant mammograms with the normal mammograms.

The evaluation metrics of each classifier evaluated by elevenfold cross-validation for the one-stage study are given in Table 5.

**Figure 4:**
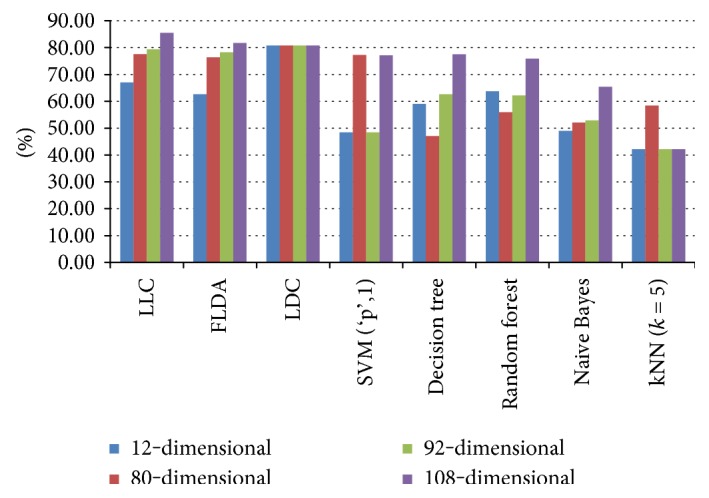
Average classification accuracies of eight different classifiers obtained by elevenfold cross-validation for the one-stage study using different feature sets.

The one-stage study is also achieved using three additional sets of feature vectors in order to demonstrate the discriminative power of the proposed 108-dimensional feature vector ensemble. These sets consist of 12-dimensional statistical feature vectors, 80-dimensional LCP-based feature vectors, and 92-dimensional feature vectors concatenated by the LCP-based with statistical features. The average classification accuracies of the classifiers for the one-stage study obtained by elevenfold cross-validation technique using different feature vector sets are shown in Figure 4. It can be inferred from Figure 4 that classification accuracies are increased when 92-dimensional feature vectors are used rather than only statistical or only LCP-based features. Furthermore, 108-dimensional feature vectors provide higher recognition accuracies than the 92-dimensional feature vectors. These results obviously prove the effectiveness of the proposed feature ensemble.

#### 3.1.2. Two-Stage Study

**Figure 5:**
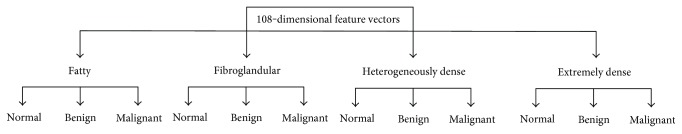
Flowchart designed for the two-stage study.

The recognition accuracy for breast cancer diagnosis is expected to be enhanced by the two-stage study, which is composed of the breast tissue and health status classification. In the first stage of this study, the feature vectors are classified into breast tissue classes (fatty, fibroglandular, heterogeneously dense, and extremely dense). Then, the breast-tissue-type-defined feature vectors are classified into normal, benign, and malignant classes in the second stage. The flowchart for the two-stage study is shown in Figure 5.

**Figure 6:**
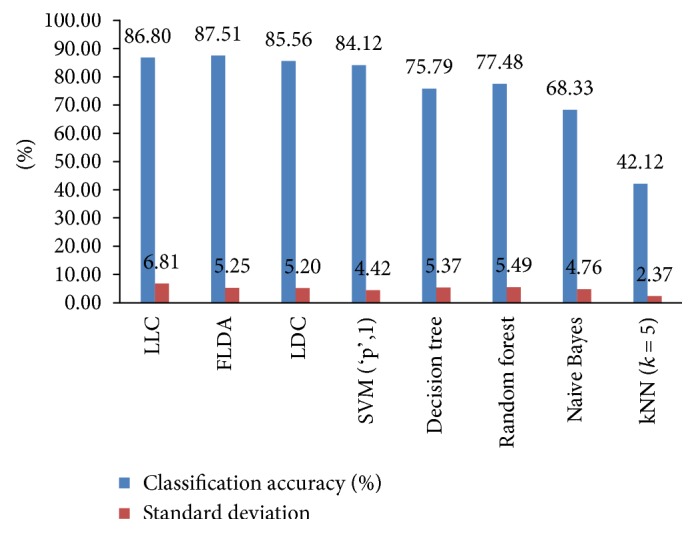
Average classification accuracies and standard deviations of classifiers obtained by elevenfold cross-validation for the two-stage study.

The average classification accuracies and standard deviations of classifiers obtained by elevenfold cross-validation technique for the two-stage study are shown in Figure 6. A maximum of 87.51% accuracy rate is attained using the FLDA classifier among eight well-known classifiers. For this case, the LLC classifier performs worse than FLDA classifier as the prior probabilities of the classes are no longer equal.

As it can be explicitly inferred from Figure 6, the top three classifiers based on performance are the FLDA, LLC, and LDC. The results of these classifiers are combined via a majority voting technique to increase the classification accuracy to 88.79%.

The total confusion matrices of the (a) FLDA, (b) LLC, and (c) LDC classifiers obtained by elevenfold cross-validation for the two-stage study and the total confusion matrix of the classifier combination obtained by elevenfold cross-validation for the two-stage study are given in Tables 6 and 7, respectively. Similar results are obtained in the two-stage study as in the one-stage study. The confusion matrices in Tables 6 and 7 clearly show that the false negatives and false positives for both benign and malignant classes belong to the normal class. The terms N., B., and M. in Table 6 refer to the normal, benign and malignant classes, respectively.

The evaluation metrics of each classifier and the classifier combination evaluated by elevenfold cross-validation for the two-stage study are given in Tables 8 and 9, respectively.

#### 3.1.3. Three-Stage Study

**Figure 7:**
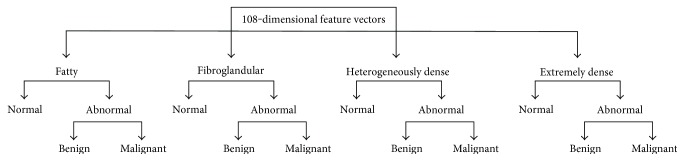
Flowchart designed for the three-stage study.

After the classification accuracies are enhanced by the two-stage study, the authors propose a three-stage study for further improvement. The three-stage study consists of both breast tissue and health status classification, where the health status classification is achieved through two consecutive stages. In the first stage of this study, the feature vectors are classified into breast tissue classes similar to those in the two-stage study. The breast-tissue-type-defined feature vectors are then categorized into normal and abnormal classes in the second stage. Finally, in the last stage, the feature vectors labeled as abnormal classes are categorized into benign and malignant classes. The flowchart for the three-stage study is illustrated in Figure 7.

**Figure 8:**
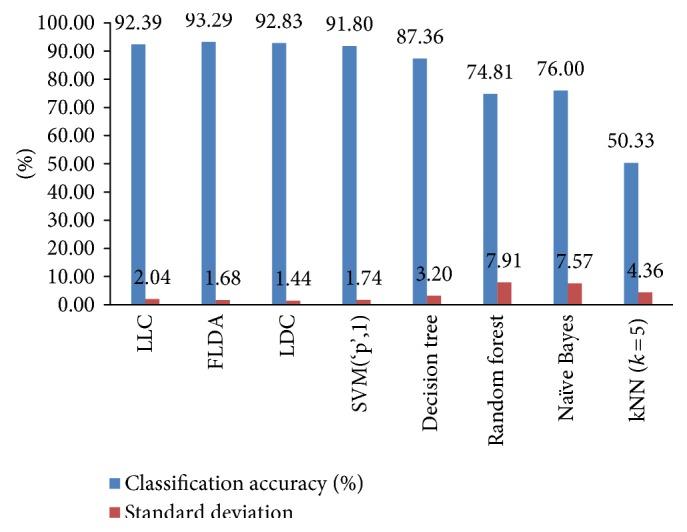
Average classification accuracies and standard deviations of classifiers obtained by elevenfold cross-validation for the three-stage study.

The average classification accuracies and standard deviations of eight classifiers obtained by elevenfold cross-validation technique for the three-stage study are graphically shown in Figure 8. The FLDA has the best classification performance with a maximum of 93.29% accuracy rate among all classifiers. In this case, as the prior probabilities of the classes are not equal again as in the two-stage study, the classification success of the LLC classifier is less than that of the FLDA and LDC classifiers.

The total confusion matrices of the (a) FLDA, (b) LDC, and (c) LLC classifiers obtained by elevenfold cross-validation for the three-stage study, and the total confusion matrix of classifier combination obtained by elevenfold cross-validation for the three-stage study are given in Tables 10 and 11, respectively. In the three-stage study, as seen in the tables, mammograms in normal and benign classes are exactly inseparable from each other, while malignant mammograms are clearly distinguished from the normal and benign classes. The terms N., B., and M. in Table 10 stand for the normal, benign, and malignant classes, respectively.

If Figure 8 is carefully examined, the FLDA, LDC, and LLC classifiers, as in the two-stage study, are the best three classifiers in terms of recognition accuracy. The results of these classifiers are combined via majority voting and eventually the classification performance is increased to 93.52%.

The evaluation metrics of each classifier and the classifier combination evaluated by elevenfold cross-validation for the three-stage study are given in Tables 12 and 13, respectively.

### 3.2. Discussion

The proposed feature ensemble is formed by concatenating the LCP-based, statistical, and frequency-domain features. The LCP algorithm is performed by itself for several image processing applications. The motivation behind the usage of the LCP algorithm for feature extraction relies on the decomposition of information existing in breast mammogram images. Moreover, the LCP features include pixel-wise relationships. As it covers relatively few relationships among pixels in a breast mammogram image, the LCP is used as the fundamental feature extraction method to explore the underlying information in an image. However, the LCP features are not completely adequate to efficiently classify mammogram parts because it can be affected by various issues. Therefore, the use of LCP only will not result in the most representative features for a mammogram. Furthermore, twelve statistical features were calculated from the LCP features. The positive impact of statistical features extracted directly from the image texture on classification success is already known [52]. In addition, the LCP feature vectors extracted from breast mammograms are indicated as successfully discriminative features [5]. Hence, in this study, the statistical features are obtained from the LCP feature vectors rather than directly from the mammogram image pixel matrices. Moreover, 16 frequency-domain features are computed and appended to other two types of features (LCP-based and statistical features). Since the brightness is one of the most significant issues for breast cancer diagnosis and the variations of brightness in a mammogram image can be obviously observed in spatial domain, it is assumed that frequency-domain features are also representative of mammograms in this study. Ultimately, the feature vectors that have more representative power and are more robust to numerous effects are constructed by this method.

Additionally, a multistage classification scheme is proposed in this study. It consists of three cases: the one-stage study, two-stage study, and three-stage study. In the one-stage study, the feature vectors are classified according to only their health status regardless of the breast tissue type of mammograms. The standard deviation values for the one-stage study are high since some folds in cross-validation process provide high recognition accuracies but the other folds give much lower classification accuracies. This situation clearly implies that the accuracy results of the one-stage study are directly related with the mammogram parts used in train/test separation of each fold. If a test set includes more similar parts compared to those in the corresponding train set, the accuracy suddenly raises. On the contrary, if the similarities between the test and train sets are weak, the classification fails. This consequence obviously reveals that the one-stage study does not give trustworthy accuracy results. In order to prevent the high standard deviation problem and increase the classification accuracy rates, the two-stage study is implemented. In the two-stage study, both breast tissue and health status classification are consecutively performed. By this way, the breast tissue types of mammograms are taken into consideration so that a more reliable classification is achieved. The trustworthiness of recognition can be inferred by examining the standard deviation values for each classifier. These values are much lower compared to those obtained in the one-stage study. Therefore, the accuracy results of the two-stage study are not related with the mammogram parts treated in train/test separation of each fold. The cross-validation process gives more reliable accuracy rates. Finally, the three-stage study considers both breast tissue and health status classification as the two-stage study does, except that the health status classification is realized through two consequent stages. By this way, the lowest standard deviation values especially for the classifiers which give higher recognition accuracies are obtained. This outcome apparently exposes that the three-stage study not only performs the most reliable classification process but also is independent from mammogram parts used in training and test sets of each cross-validation fold. Besides, the most successful experiments are achieved in the three-stage case. Ultimately, if one considers both success and reliability issues at the same time in this classification problem, the three-stage case provide these two issues simultaneously.

The mammogram parts of fatty breast tissue type in the IRMA database are classified using only LCP-based feature vectors, and a maximum of 90.60% recognition accuracy is attained in [5]. By the proposed feature ensemble and multistage classification, this accuracy is effectively increased to 93.52% for all tissue types rather than for only one breast tissue type. This result explicitly shows that the new feature ensemble is more representative than an LCP-based feature vector by itself, and the proposed multistage classification scheme is more successful and reliable than a single-stage classification for breast cancer diagnosis. The comparison of the proposed study with other studies in the literature is given in Table 14.

## 4. Conclusion

Breast cancer is the second major reason for female deaths resulting from cancer worldwide. Although there is no known way to prevent breast cancer, mortality can be reduced only with early diagnosis. Therefore, the computer-aided diagnosis (CAD) systems are very important as they allow radiologists to reconsider mammogram images with increased sensitivity of detection and diagnosis. In this study, a multistage classification scheme using a novel and discriminative feature ensemble to be implemented in a CAD system for breast cancer diagnosis is proposed. The proposed system is verified using the IRMA database. This database includes all twelve classes defined by BI-RADS, which are four different breast tissue types, and three different health status cases for each breast tissue type. The proposed feature ensemble is formed by concatenating the 80-dimensional LCP-based features obtained from the one-level, two-dimensional discrete wavelet transform of the preprocessed mammogram images, 12-dimensional statistical features computed from the LCP-based features, and 16-dimensional frequency-domain features calculated from the two-level two-dimensional discrete wavelet transform of the preprocessed mammogram images. In this study, a multistage classification scheme, namely the one-stage study, two-stage study, and three-stage study cases, is presented. The feature vectors are classified directly according to their health status in the one-stage study. In the two-stage study, the health status classification of each breast tissue type, determined in the first stage where the breast tissue classification is achieved, is executed. The three-stage study also considers both breast tissue and health status; however, in this case, the health status classification is performed with two consequent stages, where the normal and abnormal mammograms are determined first, and the abnormal defined mammograms are then classified as benign and malignant. The maximum recognition accuracy of the proposed system is obtained in the three-stage study. These results clearly indicate that using three-stage study is very effective for a CAD system and helpful for radiologists to make more accurate breast cancer diagnoses.

## Figures and Tables

**Table 1:** Statistical features and their mathematical representations.

Energy	$\overset{N}{\underset{i=1}{\sum }}X_{i}^{2}$
Mean	$\mu =\frac{1}{N}\cdot \overset{N}{\underset{i=1}{\sum }}X_{i}$
Variance	$Var=\frac{1}{N-1}\cdot \overset{N}{\underset{i=1}{\sum }}{\left(X_{i}-\mu \right)}^{2}$
Maximum	Maximum{X_*i*_|*i* = 1, 2,…, *N*}
Minimum	Minimum{X_*i*_|*i* = 1, 2,…, *N*}
Standard deviation	$\sigma =\sqrt{\mathrm{var}}$
Skewness	$\frac{1}{\sigma^{3}}\cdot \overset{N}{\underset{i=1}{\sum }}{\left(X_{i}-\mu \right)}^{3}$
Kurtosis	$\frac{1}{\sigma^{4}}\cdot \overset{N}{\underset{i=1}{\sum }}{\left(X_{i}-\mu \right)}^{4}$
Area descriptor [50]	$\frac{\sigma }{\mu }$
Mean energy	$\mu_{Energy}=\frac{1}{N}\cdot \overset{N}{\underset{i=1}{\sum }}X_{i}^{2}$
Energy variance	$\frac{1}{N-1}\cdot \overset{N}{\underset{i=1}{\sum }}{\left(X_{i}^{2}-\mu_{Energy}\right)}^{2}$
Entropy	$-\overset{N}{\underset{i=1}{\sum }}p\left(X_{i}\right)\cdot {log}_{2}p\left(X_{i}\right)$

**Table 2:** Feature vector construction process.

108-dimensional feature vector content		
LCP-based feature vector	Statistical features	Frequency-domain features
80 × 1	12 × 1	16 × 1
The 80-dimensional LCP-based feature vector		LLLL: energy
		LLLH: energy
	LCP: energy	LLHL: energy
	LCP: mean	LLHH: energy
	LCP: variance	LHLL: energy
	LCP: maximum	LHLH: energy
	LCP: minimum	LHHL: energy
	LCP: standard deviation	LHHH: energy
	LCP: skewness	HLLL: energy
	LCP: kurtosis	HLLH: energy
	LCP: area descriptor	HLHL: energy
	LCP: mean energy	HLHH: energy
	LCP: energy variance	HHLL: energy
	LCP: entropy	HHLH: energy
		HHHL: energy
		HHHH: energy

**Table 3:** Evaluation metrics and their mathematical representations.

TP: true positive	TN: true negative
FP: false positive	FN: false negative
Sensitivity (SNS)	$\%SNS=\frac{TP}{TP+FN}\cdot 100$
Specificity (SPC)	$\%SPC=\frac{TN}{TN+FP}\cdot 100$
Positive predictive value (PPV)	$\%PPV=\frac{TP}{TP+FP}\cdot 100$
Negative predictive value (NPV)	$\%NPV=\frac{TN}{TN+FN}\cdot 100$
False-positive rate (FPR)	$\%FPR=\frac{FP}{FP+TN}\cdot 100$
False-negative rate (FNR)	$\%FNR=\frac{FN}{TP+FN}\cdot 100$
False-discovery rate (FDR)	$\%FDR=\frac{FP}{TP+FP}\cdot 100$
False omission rate (FOR)	$\%FOR=\frac{FN}{TN+FN}\cdot 100$
Accuracy (ACC)	$\%ACC=\frac{TP+TN}{TP+FP+TN+FN}\cdot 100$

**Table 4:** Total confusion matrix of the LLC classifier obtained by elevenfold cross-validation for the one-stage study.

		Predicted classes		
		Normal	Benign	Malignant
Actual classes	Normal	862	91	59
	Benign	123	889	0
	Malignant	166	2	844

**Table 5:** The evaluation metrics of each classifier evaluated by elevenfold cross-validation for the one-stage study.

Classifier	SNS	SPC	PPV	NPV	FPR	FNR	FDR	FOR	ACC
LLC	85.47	92.74	85.47	92.74	7.26	14.53	14.53	7.26	85.47
FLDA	81.72	90.86	81.72	90.86	9.14	18.28	18.28	9.14	81.72
LDC	80.80	90.40	80.80	90.40	9.60	19.20	19.20	9.60	80.80
SVM (‘p', 1)	77.14	88.57	77.14	88.57	11.43	22.86	22.86	11.43	77.14
Decision tree	77.50	88.75	77.50	88.75	11.25	22.50	22.50	11.25	77.50
Random forest	75.86	87.93	75.86	87.93	12.07	24.14	24.14	12.07	75.86
Naïve Bayes	65.42	82.71	65.42	82.71	17.29	34.58	34.58	17.29	65.42
kNN (*k* = 5)	42.13	71.06	42.13	71.06	28.94	57.87	57.87	28.94	42.13

**(a):** 

		Predicted classes		
		N.	B.	M.
Actual classes	N.	872	52	88
	B.	186	826	0
	M.	142	5	865

**(b):** 

		Predicted classes		
		N.	B.	M.
Actual classes	N.	835	105	72
	B.	135	874	3
	M.	153	0	859

**(c):** 

		Predicted classes		
		N.	B.	M.
Actual classes	N.	877	25	108
	B.	218	794	0
	M.	228	2	782

**Table 7:** Total confusion matrix of classifier combination obtained by elevenfold cross-validation for the two-stage study.

		Predicted classes		
		Normal	Benign	Malignant
Actual classes	Normal	887	41	84
	Benign	162	850	0
	Malignant	140	2	870

**Table 8:** The evaluation metrics of each classifier evaluated by elevenfold cross-validation for the two-stage study.

Classifier	SNS	SPC	PPV	NPV	FPR	FNR	FDR	FOR	ACC
LLC	86.49	92.11	84.92	91.78	7.89	13.51	15.08	8.22	86.80
FLDA	87.45	92.18	85.25	91.76	7.82	12.55	14.75	8.24	87.51
LDC	84.68	90.29	81.66	89.41	9.71	15.32	18.34	10.59	85.56
SVM (‘p', 1)	84.46	90.13	80.30	89.51	9.87	15.54	19.70	10.49	84.12
Decision tree	75.78	87.64	74.93	88.00	12.36	24.22	25.07	12.00	75.79
Random forest	77.51	88.29	77.38	88.38	11.71	22.49	22.62	11.62	77.48
Naïve Bayes	67.81	83.86	71.38	84.75	16.14	32.19	28.62	15.25	68.33
kNN (*k* = 5)	42.13	70.51	40.73	70.81	29.49	57.87	59.27	29.19	42.12

**Table 9:** The evaluation metric of classifier combination evaluated by elevenfold cross-validation for the two-stage study.

Classifier	SNS	SPC	PPV	NPV	FPR	FNR	FDR	FOR	ACC
Classifier combination	88.67	92.93	86.32	92.45	7.07	11.33	13.68	7.55	88.79

**(a):** 

		Predicted classes		
		N.	B.	M.
Actual classes	N.	393	445	174
	B.	174	629	209
	M.	139	260	613

**(b):** 

		Predicted classes		
		N.	B.	M.
Actual classes	N.	382	490	140
	B.	183	697	132
	M.	204	311	497

**(c):** 

		Predicted classes		
		N.	B.	M.
Actual classes	N.	336	467	209
	B.	145	624	243
	M.	158	249	605

**Table 11:** Total confusion matrix of classifier combination obtained by elevenfold cross-validation for the three-stage study.

		Predicted classes		
		Normal	Benign	Malignant
Actual classes	Normal	393	444	175
	Benign	174	630	208
	Malignant	139	257	616

**Table 12:** The evaluation metrics of each classifier evaluated by elevenfold cross-validation for the three-stage study.

Classifier	SNS	SPC	PPV	NPV	FPR	FNR	FDR	FOR	ACC
LLC	87.90	93.25	90.73	94.05	6.75	12.10	9.27	5.95	92.39
FLDA	87.91	92.90	90.61	93.96	7.10	12.09	9.39	6.04	93.29
LDC	87.30	91.71	88.29	92.17	8.29	12.70	11.71	7.83	92.83
SVM (‘p', 1)	74.85	85.29	79.66	88.45	14.71	25.15	15.43	11.55	91.80
Decision tree	85.68	91.60	87.03	92.18	8.40	14.32	12.97	7.82	87.36
Random forest	79.54	88.69	78.44	89.24	11.31	20.46	21.56	10.76	74.81
Naïve Bayes	65.23	82.04	68.49	85.73	17.96	34.77	31.51	14.27	76.00
kNN (*k* = 5)	63.02	77.98	54.81	78.50	22.02	36.98	45.19	21.50	50.33

**Table 13:** The evaluation metric of classifier combination evaluated by elevenfold cross-validation for the three-stage study.

Classifier	SNS	SPC	PPV	NPV	FPR	FNR	FDR	FOR	ACC
Classifier combination	87.91	92.90	90.61	93.96	7.10	12.09	9.39	6.04	93.52

**Table 14:** The comparison of the results for the proposed CAD system.

Authors	Features	Number of images	Accuracy
Ganesan et al. [21]	Statistical features	300	91%
Korkmaz and Korkmaz [65]	Statistical features	378	98.3%
Vikhe and Thool [66]	Unstated	130	91%
Jen and Yu [67]	Statistical and gradient features	322	86%
Vadivel and Surendiran [68]	Shape and margin	224	87.76%
Acharya et al. [69]	Area, homogeneity	360	88.80%

## References

[1] American Cancer Society (2015). *Cancer Facts & Figures 2015*.

[2] American Cancer Society (2009). *Cancer Facts & Figures 2009*.

[3] Jemal A., Bray F., Center M. M., Ferlay J., Ward E., Forman D. (2011). Global cancer statistics. *CA: a Cancer Journal for Clinicians*.

[4] American Cancer Society (2013). *Breast Cancer Facts & Figures 2013-2014*.

[5] Ergin S., Kılınç O. (2014). A new feature extraction framework based on wavelets for breast cancer diagnosis. *Computers in Biology and Medicine*.

[6] Birdwell R. L., Ikeda D. M., O’Shaughnessy K. F., Sickles E. A. (2001). Mammographic characteristics of 115 missed cancers later detected with screening mammography and the potential utility of computer-aided detection. *Radiology*.

[7] Al-Ghaib H., Adhami R., Scott M. (2016). An overview of mammogram analysis. *IEEE Potentials*.

[8] Al-Najdawi N., Biltawi M., Tedmori S. (2015). Mammogram image visual enhancement, mass segmentation and classification. *Applied Soft Computing*.

[9] George Y. M., Zayed H. H., Roushdy M. I., Elbagoury B. M. (2014). Remote computer-aided breast cancer detection and diagnosis system based on cytological images. *IEEE Systems Journal*.

[10] Tai S. C., Chen Z. S., Tsai W. T. (2014). An automatic mass detection system in mammograms based on complex texture features. *Journal of Biomedical and Health Informatics*.

[11] Yang Z., Dong M., Guoa Y. (2016). A new method of micro-calcifications detection in digitized mammograms based on improved simplified PCNN. *Neurocomputing*.

[12] Anitha J., Dinesh Peter J. (2015). Mammogram segmentation using maximal cell strength updation in cellular automata. *Medical & Biological Engineering & Computing*.

[13] Husaain M. (2014). Mammogram enhancement using lifting dyadic wavelet transform and normalized Tsallis entropy. *Journal of Computer Science and Technology*.

[14] Silva J. N., Filho A. O. C., Silva A. C., Paiva A. C., Gattass M. (2015). Automatic detection of masses in mammograms using quality threshold clustering, correlogram function, and SVM. *Journal of Digital Imaging*.

[15] Gupta B., Tiwari M. (2016). A tool supported approach for brightness preserving contrast enhancement and mass segmentation of mammogram images using histogram modified grey relational analysis. *Multidimensional Systems and Signal Processing*.

[16] Lado M. J., Cadarso-Suárez C., Roca-Pardiñas J., Tahoces P. G. (2008). Categorical variables, interactions and generalized additive models. Applications in computer-aided diagnosis systems. *Computers in Biology and Medicine*.

[17] Kekre H. B., Sarode T., Gharge S. (2009). Tumor detection in mammography images using vector quantization technique. *International Journal of Intelligent Information Technology Application*.

[18] Kekre H. B., Sarode T., Gharge S., Raut K. (2010). Detection of cancer using vector quantization for segmentation. *International Journal of Computers and Applications*.

[19] Haider W., Sharif M., Raza M. (2011). Achieving accuracy in early stage tumor identification systems based on image segmentation and 3d structure analysis. *Computer Engineering and Intelligent Systems*.

[20] Radovic M., Djokovic M., Peulic A., Filipovic N. Application of data mining algorithms for mammogram classification.

[21] Ganesan K., Acharya U. R., Chua C. K., Min L. C., Matthew B., Thomas A. K. (2013). Decision support system for breast cancer detection using mammograms. *Proceedings of the Institution of Mechanical Engineers. Part H*.

[22] Li J. B., Wang Y. H., Chu S. C., Roddick J. F. (2014). Kernel self-optimization learning for kernel-based feature extraction and recognition. *Information Sciences*.

[23] Ramos R. P., do Nascimento M. Z., Pereira D. C. (2012). Texture extraction: an evaluation of ridgelet, wavelet and co-occurrence based techniques applied to mammograms. *Expert Systems with Applications*.

[24] Shradhananda B., Banshidhar M., Ratnakar D. (2015). Mammogram classification using two dimensional discrete wavelet transform and gray-level co-occurrence matrix for detection of breast cancer. *Neurocomputing*.

[25] Vallez N., Bueno G., Deniz O. (2013). Breast density classification to reduce false positives in CADe systems. *Computer Methods and Programs in Biomedicine*.

[26] Biswas S. K., Mukherjee D. P. (2011). Recognizing architectural distortion in mammogram: a multiscale texture modeling approach with GMM. *IEEE Transactions on Biomedical Engineering*.

[27] Pal N. R., Bhowmick B., Patel S. K., Pal S., Das J. (2008). A multistage neural network aided system for detection of microcalcifications in digitized mammograms. *Neurocomputing*.

[28] Chen Z., Strange H., Oliver A., Denton E. R. E., Boggis C., Zwiggelaar R. (2015). Topological modeling and classification of mammographic microcalcification clusters. *IEEE Tractions on Bio-Medical Engineering*.

[29] Papadopoulos A., Fotiadis D. I., Costaridou L. (2008). Improvement of microcalcification cluster detection in mammography utilizing image enhancement techniques. *Computers in Biology and Medicine*.

[30] Agrawal P., Vatsa M., Singh R. (2014). Saliency based mass detection from screening mammograms. *Signal Processing*.

[31] Moayedi F., Azimifar Z., Boostani R., Katebi S. (2010). Contourlet-based mammography mass classification using the SVM family. *Computers in Biology and Medicine*.

[32] Karahaliou A. N., Boniatis I. S., Skiadopoulos S. G. (2008). Breast cancer diagnosis: analyzing texture of tissue surrounding microcalcifications. *IEEE Transactions on Information Technology in Biomedicine*.

[33] Sampaio W. B., Diniz E. M., Silva A. C., de Paiva A. C., Gattass M. (2011). Detection of masses in mammogram images using CNN, geostatistic functions and SVM. *Computers in Biology and Medicine*.

[34] Keleş A., Keleş A., Yavuz U. (2011). Expert system based on neuro-fuzzy rules for diagnosis breast cancer. *Expert Systems with Applications*.

[35] Krishnan M. M. R., Banerjee S., Chakraborty C., Chakraborty C., Ray A. K. (2010). Statistical analysis of mammographic features and its classification using support vector machine. *Expert Systems with Applications*.

[36] Verma B., McLeod P., Klevansky A. (2009). A novel soft cluster neural network for the classification of suspicious areas in digital mammograms. *Pattern Recognition*.

[37] Oliver A., Torrent A., Liado X. (2012). Automatic microcalcification and cluster detection for digital and digitized mammograms. *Knowledge-Based Systems*.

[38] Zhang X., Gao X. (2012). Twin support vector machines and subspace learning techniques for microcalcification clusters detection. *Engineering Applications of Artificial Intelligence*.

[39] Ganesan K., Acharya U. R., Chua C. K., Lim C. M., Abraham K. T. (2014). One-class classification of mammograms using trace transform functions. *IEEE Transactions on Instrumentation and Measurement*.

[40] Malar E., Kandaswamy A., Chakravarthy D., GiriDharan A. (2012). A novel approach for detection and classification of mammographic microcalcifications using wavelet analysis and extreme learning machine. *Computers in Biology and Medicine*.

[41] Bria A., Karssemeijer N., Tortorella F. (2014). Learning from unbalanced data: a cascade-based approach for detecting clustered microcalcifications. *Medical Image Analysis*.

[42] Hachama M., Desolneux A., Richard F. J. P. (2012). Bayesian technique for image classifying registration. *IEEE Transactions on image processing*.

[43] Savitha R., Suresh S., Sundararajan N. (2013). Projection-based fast learning fully complex-valued relaxation neural network. *IEEE Transactions on Neural Networks and Learning Systems*.

[44] Ventura J. A., Chen J. M. (1992). Segmentation of two-dimensional curve contours. *Pattern Recognition*.

[45] Guliato D., Rangayyan R. M., Carvalho J. D., Santigo S. A. (2008). Polygonal modeling of contours of breast tumors with the preservation of spicules. *IEEE Transactions on Biomedical Engineering*.

[46] Buades A., Coll B., Morel J. M. (2005). A review of image denoising algorithms, with a new one. *Multiscale Modeling & Simulation*.

[47] Guo Y., Zhao G., Pietkäinen M. (2011). Texture classification using a linear configuration model based descriptor. *British Machine Vision Association*.

[48] Işıklı Esener İ., Ergin S., Yüksel T. A new ensemble of features for breast cancer diagnosis.

[49] Deserno T. M., de Oliveira J. E. E., Araujo A. A. Towards computer-aided diagnostics of screening mammography using content-based image retrieval.

[50] Ahonen T., Hadid A., Pietkäinen M. (2006). Face description with local binary patterns: application to face recognition. *IEEE Transactions on Pattern Analysis and Machine Intelligence*.

[51] Zhang B., Gao Y., Zhao S., Liu J. (2010). Local derivative pattern versus local binary pattern: face recognition with high-order local pattern descriptor. *IEEE Biometrics Compendium*.

[52] Zhang G., Huang X., Li S. Z., Wang Y., Wu X. (2005). Boosting local binary pattern (LBP)-based face recognition. *Lecture Notes in Computer Science*.

[53] Ojala T., Pietkäinen M., Mäenpää T. (2002). Multiresolution gray-scale and rotation invariant texture classification with local binary patterns. *IEEE Transactions on Pattern Analysis and Machine Intelligence*.

[54] Woods K., Doss C., Bowyer K., Solka J., Priebe C., Philip W. (1993). Comparative evaluation of pattern recognition techniques for detection of microcalcifications in mammography. *International Journal of Pattern Recognition and Artificial Intelligence*.

[55] Daubechies I. Ten lectures on wavelets.

[56] Fisher R. A. (1936). The use of multiple measurements in taxonomic problems. *Annals of Eugenics*.

[57] Webb A. R. (2002). Linear discriminant analysis. *Statistical Pattern Recognition*.

[58] Chapelle O., Haffner P., Vapnik V. N. (1999). Support vector machines for histogram-based image classification. *IEEE Transactions on Neural Networks*.

[59] Özkan K., Ergin S., Işık Ş., Işıklı İ. (2015). A new classification scheme of plastic wastes based upon recycling labels. *Waste Management*.

[60] Webb A. R. (2002). Linear discriminant analysis. *Statistical Pattern Recognition*.

[61] Safavian S. R., Landgrebe D. (1991). A survey of decision tree classifier methodology. *IEEE Transactions on Systems, Man, and Cybernetics*.

[62] Breiman L. (2001). Random forests. *Machine Learning*.

[63] Chen J., Huang H., Tian S., Qu Y. (2009). Feature selection for text classification with naive Bayes. *Expert Systems with Applications*.

[64] Fix E., Hodges J. (1989). *Discriminatory Analysis, Nonparametric Discrimination: Consistency Properties, International Statistical Review (Revue Internationale de Statistique)*.

[65] Korkmaz S. A., Korkmaz M. F. (2015). A new method based cancer detection in mammogram textures by finding feature weights and using Kullback-Leibler measure with kernel estimation. *Optik*.

[66] Vikhe P. S., Thool V. R. (2016). Mass detection in mammographic images using wavelet processing and adaptive threshold technique. *Journal of Medical Systems*.

[67] Jen C. C., Yu S. S. (2015). Automatic detection of abnormal mammograms in mammographic images. *Expert Systems with Applications*.

[68] Vadivel A., Surendiran B. (2013). A fuzzy rule-based approach for characterization of mammogram masses into BI-RADS shape categories. *Computers in Biology and Medicine*.

[69] Acharya U. R., Ng E. Y. K., Hong Y., Jie Y., Kaw G. J. L. (2008). Computer-based identification of breast cancer using digitized mammograms. *Journal of Medical Systems*.

